# Supplementary material for: The Impact of Temperature and Ethanol Concentration on the Global Recovery of Specific Polyphenols in an Integrated HPLE/RP Process on Carménère Pomace Extracts
Source: Molecules. 2019 Aug 29;24(17):3145. doi: 10.3390/molecules24173145 (PMC6749334; doi:10.3390/molecules24173145)
Supplement: Supplementary file 1 [file molecules-24-03145-s001.pdf]

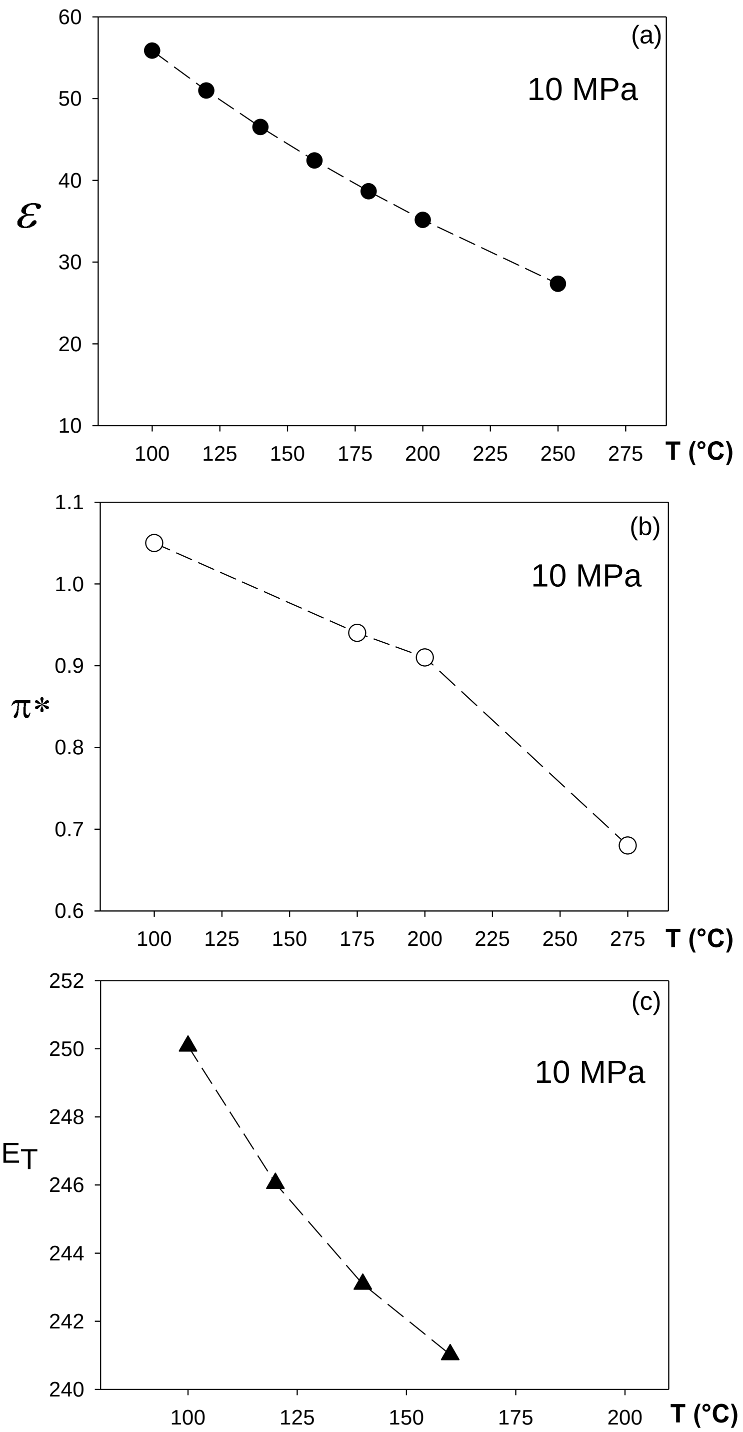

Figure S1. Water polarity expressed as dielectric constant (Ɛ), polarity/polarizability (π*) and Reichardt's scale (E_T) at pressure constant (10 MPa).

Adapted from Arched et al. (1989); Alghoul et al. (2017); Jessop et al. (2012); Lu et al. (2002)

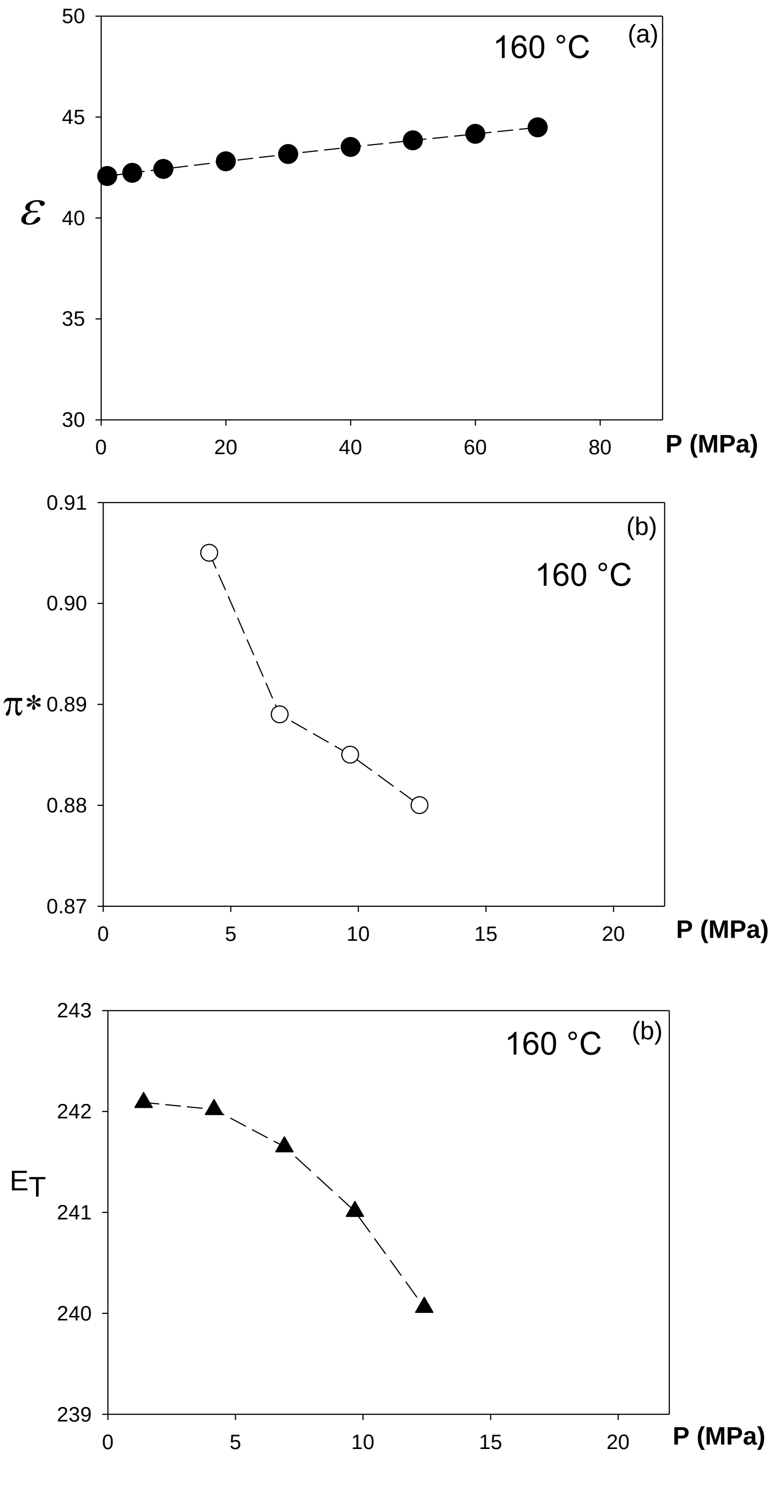

Figure S2. Water polarity expressed as dielectric constant (Ɛ), polarity/polarizability (π*) and Reichardt's scale (E_T) at temperature constant (160°C).

Adapted from Arched et al. (1989); Alghoul et al. (2017)
